# Ductal carcinoma *in situ* of the breast, a population-based study of epidemiology and pathology

**DOI:** 10.1038/sj.bjc.6601677

**Published:** 2004-02-24

**Authors:** A Kricker, C Goumas, B Armstrong

**Affiliations:** 1 School of Public Health, Level 6, Medical Foundation Building K25, University of Sydney NSW 2006, Australia; 2 School of Public Health, Edward Ford Building A27, University of Sydney NSW 2006, Australia

**Keywords:** DCIS, epidemiology, pathology, population-based

## Abstract

In a population-based series of 2109 women with ductal carcinoma *in situ* (DCIS) diagnosed in 1995–2000 in New South Wales, Australia, incidence increased by an average of 5.5% a year, mostly between 1995 and 1996 and in women 50–69 years of age. This increase paralleled the increases in mammographic screening. BreastScreen NSW, an organised mammographic screening programme, detected 65% of all DCIS. High-grade lesions were 54% of all lesions and were more likely to be 2+ cm in diameter (OR=2.12, 95%CI 1.46–3.14) than low-grade lesions. In all, 40% of DCIS in women younger than 40 years was 2+ cm in diameter compared with 21% in women 40 years and older. Young age, high grade, mixed architecture and multifocality were significant and independent predictors of 2+ cm DCIS.

The epidemiology of ductal carcinoma *in situ* (DCIS) has yet to be described adequately. While mammographic screening has undoubtedly caused increasing diagnosis of DCIS (Ernster *et al*, 1996,^2002^; Levi *et al*, 1997; Barchielli *et al*, 1999), studies to date have mainly been small (Levi *et al*, 1997; Barchielli *et al*, 1999) or described populations that had no organised screening programme (Choi *et al*, 1996; Ernster *et al*, 1996; Zheng *et al*, 1997). Population-based descriptions of epidemiology and pathology of DCIS in sizeable screened populations are nonexistent.

We describe the epidemiology and pathology of newly diagnosed DCIS in the 2.7 million female population of New South Wales (NSW) Australia in 1995–2000.

## MATERIALS AND METHODS

### Data

NSW women with a first diagnosis of DCIS in 1995–2000 and notified to the NSW Central Cancer Registry were eligible for the study; those with a previous or simultaneous (same month) diagnosis of invasive breast cancer were excluded. Ductal carcinoma *in situ* has been notifiable in NSW since 1993, and by 1997 all but 4% of DCIS cases diagnosed by pathology laboratories were notified to the Cancer Registry (unpublished data).

Two experienced Cancer Registry personnel extracted information on the type of specimen, size, grade, architecture, presence or absence of necrosis and multifocality of DCIS, and clearance and width of the margins from pathology reports (Kricker *et al*, 1999).

The frequency of mammography was obtained from reports of an organised screening programme, BreastScreen NSW (Estoesta *et al*, 2000; Productivity Commission, 2002), which began in 1991 and reached a steady state between 1995 and 2000. The numbers of bilateral mammograms reimbursed by the national health insurance scheme, Medicare, were also available for 1995–99 (http://www.hic.gov.au/provider s/health_statistics/statistica l_reporting/medicare.htm). These mammograms would include an unknown but not high proportion of mammograms that were primarily diagnostic. BreastScreen and Medicare account for most of the screening mammography in Australia.

### Analyses

Incidence rates and 95% confidence intervals (CIs) (Dobson *et al*, 1991) were calculated in 5- (DCIS) or 10-year (mammography) age groups and age-standardised to the World population. The annual percentage changes in rates were estimated in negative binomial models with terms for age group and year of diagnosis.

Cases were allocated to urban or rural areas using BreastScreen's classification (Estoesta *et al*, 2000) and to five socioeconomic (SES) groups (Australian Bureau of Statistics, 1998), and variation among them tested in Poisson regression models.

The heterogeneity of DCIS distributions by size and grade among age groups and type of architecture among years of diagnosis was evaluated by standard *χ*^2^ tests. Age, grade, architecture, multifocality and presence of necrosis were examined as predictors of size (<2 cm, 2+ cm) in logistic regression models that included year of diagnosis; the additional effects of urban or rural residence and SES of the women were also examined.

## RESULTS

### Incidence

In 1995–2000, 2109 NSW women were notified with DCIS. More than half (54%) were 50–69 years of age, the target age group for breast cancer screening in NSW, and had the highest incidence (32.3 per 100 000) (Table 1

**Table 1:** Incidence of ductal carcinoma *in situ* of the breast in NSW women in 1995–2000 by age, urban or rural residence and socioeconomic status

	**Number**	**Rate^a^**	**95% CI**
All NSW women	2109	8.6	8.2–9.0
			
*Age group*			
20–39	91	1.4	1.1–1.7
40–49	458	17.3	15.7–19.0
50–59	634	31.8	29.4–34.4
60–69	495	32.8	29.9–35.8
70–79	354	28.8	25.8–32.0
80+	77	10.6	8.4–13.3
			
50–69	1129	32.2	30.4–34.2
70+	431	24.2	21.9–26.7
			
*Urban & rural area*			
All areas	2100	8.5	8.2–8.9
Urban	1712	9.0	8.6–9.4
Rural	388	7.1	6.3–7.8
			*P*<0.001^b^
			
*Socioeconomic status (Sydney Statistical Division only)*			
All areas	1369	9.2	8.7–9.7
1 lowest	205	7.2	6.2–8.2
2	197	7.5	6.4–8.6
3	226	8.5	7.4–9.8
4	342	10.9	9.7–12.1
5 highest	399	11.2	10.1–12.4
			*P*<0.001^c^

a Rates are per 100 000 women age-standardised to the World population.

b *χ*^2^ test for difference in rates between urban and rural (metro *vs* non-metro) – Poisson regression model.

c *P*-value for heterogeneity and trend <0.001.

).

**Figure 1:**
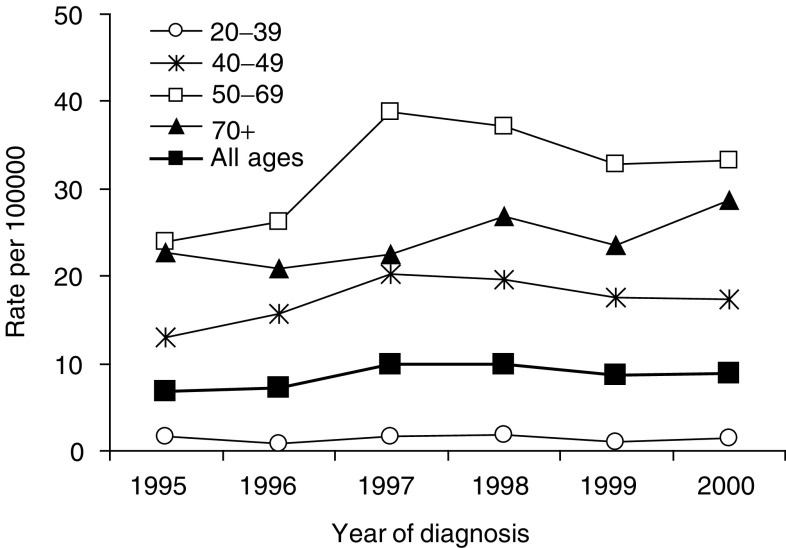
Trends in incidence of DCIS by age group in NSW women from 1995 to 2000. Rates were standardised by 5-year age intervals within broad age groups, using the World standard population.

Incidence at all ages increased from 1995 (6.8 per 100 000) to 2000 (8.9 per 100 000) (annual average 5.5%, 95% CI 2.5–8.6), mostly between 1996 and 1997 (39% increase) and in women 50–69 years of age (48% increase 1996–1997) (Figure 1

). Ductal carcinoma *in situ* rates were higher in 1998–2000 than 1995–1997 in every age group, but did not continue to increase except, perhaps, in women 70+ years of age.

The incidence of DCIS was about 25% higher in urban than rural areas of NSW (*P*<0.001) and increased strongly with increasing socio-economic status in Sydney (*P*<0.001, Table 1), but not other areas of the State (*P*=0.09). BreastScreen NSW detected 65% of incident DCIS, with higher proportions in women older than 50 years.

### Screening

**Figure 2:**
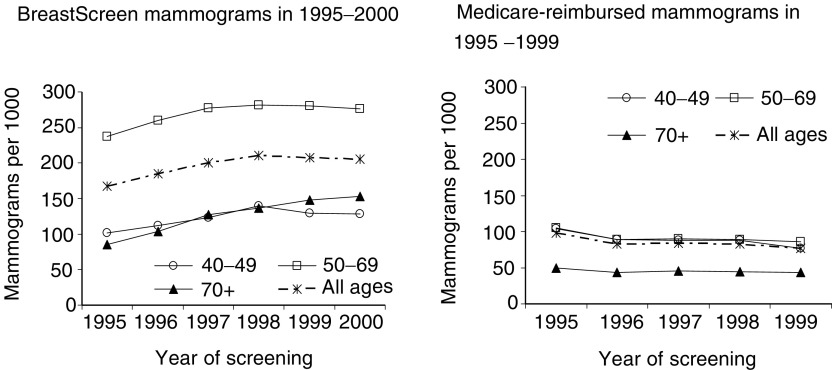
Mammogram rates in NSW women by age group – age-standardised in 10-year age groups to the World population.

BreastScreen and Medicare screened 265.1 per 1000 women 40 years of age and older in 1995 and 284.6 in 1999; all the increase was in BreastScreen. BreastScreen screened twice as many women 50–69 years of age (269.2 per 1000) as women 40–49 (122.4) and 70+ years (120.1) (Figure 2

); Medicare screening rates in the two younger age groups were nearly equal. There was little difference in the screening rates between urban (189.0 per 1000) and rural (186.6) areas.

The numbers of DCIS detected were strongly correlated with the numbers of women screened by BreastScreen in 1995–2000 (*R*=0.83, *P*<0.001). There was a weaker correlation between Medicare reimbursed mammograms and DCIS not detected by BreastScreen in 1995–1999 (*R*=0.64, *P*=0.002).

### Pathology information

Pathology reports were available for all but two cases of DCIS, mainly from excision or re-excision specimens (73%) and mastectomies (24%). Size was reported in 76%, grade in 89% (both in 69%), presence or absence of necrosis in 63%, architecture in 82% and clearance of the margins in 80%; 37% had all these items. The width of margins was reported in only 41%. All these items except the width of margins were appreciably more complete in 1998–2000 than in 1995–97.

More than half (54%) the DCIS diagnosed in 1995–2000 were high grade and 39% were 2 cm or larger (Table 2

**Table 2:** Number, percent and rate of DCIS by grade and size in NSW in 1995–2000

	**Number**	**%**	**Rate^a^**	**95% CI**
*Grade*				
Low	313	16.7	1.2	(1.1–1.4)
Intermediate	542	29.0	2.2	(2.0–2.4)
High	1015	54.3	4.2	(3.9–4.5)
Total	1870	100.0	8.6	(8.2–9.0)
Unknown	239		0.9	(0.8–1.1)
				
*Size*				
0–0.9 cm	505	31.5	2.1	(1.9–2.3)
1–1.9 cm	480	30.0	2.0	(1.8–2.2)
2–2.9 cm	278	17.4	1.1	(1.0–1.3)
3+cm	339	21.2	1.4	(1.2–1.5)
Total	1602	100.0	8.6	(8.2–9.0)
Unknown	507		2.0	(1.9–2.2)

^a^Rates are per 100 000 women age-standardised to the World population.

). More DCIS were high grade at 20–39 and 50–69 years (57%) than at other ages (50%) (*P*=0.02) and a higher proportion were 2+ cm at 20–39 (53%) than 40 years and older (38%) (*P*=0.01). Size, however, was not stated for more DCIS at 20–39 years (34%) than other ages (24%).

Most (65%) of the DCIS were described as cribriform or solid, or a mixture in which these two types predominated. Fewer were identified as comedocarcinoma (10%) in 1998–2000 than in 1995–1997 (26%) and more as a mixture of types (45% compared with 23%; *P* for difference between the two periods <0.001). High-grade DCIS were distributed across all types (mixed 32%, comedocarcinoma 27%, other specific types 21%, no specific type 20%). Most of the 2+ cm DCIS were mixed (45%) or other specific types (27%) and few were comedocarcinoma (18%) or no specific type (9%).

Age, grade, architecture and multifocality were significant independent predictors of DCIS 2+ cm in size in a logistic regression model (Table 3

**Table 3:** Association of 2+ cm DCIS with year of diagnosis, age, size, grade, necrosis and multifocality in NSW women in 1995–2000

	**<2 cm**	**2+ cm**	**OR**	**(95% CI)**	***P*-value**
*Year of diagnosis*					
1995	112	46	1		
1996	120	89	1.66	(1.05–2.64)	
1997	190	106	1.19	(0.76–1.86)	
1998	209	105	0.74	(0.47–1.18)	
1999	159	129	1.25	(0.78–1.98)	
2000	184	135	1.05	(0.66–1.67)	0.002
					
*Age group*					
20–39	28	32	1.87	(1.07–3.27)	
40–49	210	132	1.07	(0.82–1.40)	
50–69	556	316	1		
70+	180	130	1.39	(1.05–1.84)	0.03
					
*Grade*					
Low	156	58	1		
Medium	275	147	1.27	(0.86–1.87)	
High	442	365	2.14	(1.46–3.14)	
Not given	101	40	1.3	(0.78–2.17)	<0.0001
					
*Necrosis*					
Present	538	411	1		
Absent	72	45	0.94	(0.61–1.46)	
Not given	364	154	0.65	(0.49–0.86)	0.006
					
*Architecture*					
Comedocarcinoma	163	112	1		
Mixed types^a^	309	278	1.46	(1.05–2.04)	
Other specified types^b^	326	166	0.89	(0.63–1.26)	
Type unspecified	176	54	0.49	(0.32–0.73)	<0.0001
					
*Multifocal*					
No	737	377	1		
Yes	237	233	1.92	(1.52–2.43)	<0.0001

a Mixed types: cribriform with solid or papillary architecture or both (64% of mixed types); comedocarcinoma and other type (18%); other (18%).

b Other specified types: cribriform (41%), solid (40%), micropapillary (13%) and intracystic papillary carcinoma *in situ* (6%).

). Two-fold higher odds of having 2+ cm DCIS appeared to lie with a young age (20–39 years), with high grade and with multifocal lesions. The excess risks of 2+ cm DCIS also lay with mixed types of architecture (OR 1.5) compared to comedocarcinoma and diagnosis in 1996 (OR 1.7), while a report that did not mention necrosis was associated with lower odds (OR 0.7) of a larger DCIS. Otherwise, there was no evident trend across these variables. Neither place of residence (urban or rural) nor SES was significantly predictive of 2+ cm DCIS or appreciably affected the above odds ratios when added to the model.

## DISCUSSION

The main strengths of this study are its population base, detail and recency. Population-based registration of DCIS in NSW began 2 years before the first year of our study, and the study's first year coincided with that of complete population coverage by BreastScreen. Its main weakness is lack of linkage between the data sources (BreastScreen, Medicare and the Cancer Registry), without which we cannot fully describe the contribution of screening to the occurrence and outcomes of DCIS.

Other countries have observed increasing incidence of DCIS, sometimes three- to four-fold, with increasing mammographic screening (Ernster *et al*, 1996; Levi *et al*, 1997; Zheng *et al*, 1997; Barchielli *et al*, 1999; Blanks *et al*, 2000). This increase has stopped in NSW and we would expect further increases only with growth in BreastScreen participation beyond 53–54% in 1998–2000 (Estoesta *et al*, 2000; Productivity Commission, 2002) or resurgence in mammographic screening reimbursed by Medicare.

We observed a higher proportion of high-grade lesions (54%) than in Sweden (43%) and Switzerland (46%) in the early 1990s, but similar to that, 55%, in an Australia-wide sample survey (Levi *et al*, 1997; Wärnberg *et al*, 1999; Shugg *et al*, 2002). This apparent difference between Australia and these European countries may be real and due to the extent of high-grade disease, or caused by differences in the reporting of grade. It could reflect, too, differences in mammography rates since high-grade DCIS is said to show abnormal mammographic features more frequently than low grade (Evans *et al*, 2001).

We found that age, high-grade lesions, mixed architecture and multifocality significantly and independently predicted DCIS larger than 2 cm diameter. That women 20–39 years of age were more likely to have 2+ cm diameter DCIS (average 28 mm in multivariate models in this study) than older women (average 18 mm) is probably due to their lower mammography rate. That high-grade lesions were larger probably reflects a correlation with higher growth rate (1.8 mm per year low grade, 4.2 mm intermediate and 7.1 mm high grade; Thomson *et al*, 2001), particularly since they may also be more readily detectable mammographically (Evans *et al*, 2001). The Van Nuys prognostic index has shown a parallel increase of size with grade although patients treated with mastectomy, and thus probably the larger lesions, were excluded (Silverstein, 2003). Another series showed a step down from 20 mm diameter poorly differentiated DCIS to 15 mm for all other grades (Solin *et al*, 1991). Our study appears to give the first population-based estimates: the average size increased steadily from 16 mm for low grade to 20 mm for intermediate and 27 mm for high-grade DCIS in the multivariate models of Table 3. Variation in growth rate might also underlie the significant, independent association of architecture with tumour size.

The pathology reports from 1998 to 2000 reported DCIS substantially more completely than did earlier Australian reports (Kricker *et al*, 1999; Giles *et al*, 2001; Shugg *et al*, 2002). The Australian Cancer Network addressed pathology reporting of DCIS with extensive consultation among pathologists in the mid-1990s and published recommendations in 1997 (Australian Cancer Network Working Party, 1997) and 2001 (Australian Cancer Network Working Party, 2001). Their adoption by pathologists may explain the more complete reporting we observed.

Increasing incidence of DCIS is an outcome of mammographic screening for breast cancer. The high proportion of high-grade lesions we have observed suggests that its detection could contribute to reducing breast cancer mortality. If it does not, the high frequency of DCIS in association with screening may be source of unnecessary morbidity and cost. More research is needed into the costs and benefits of detection of DCIS in breast cancer screening.

## References

[bib1] Australian Bureau of Statistics (1998) Census of Population and Housing: Socio-Economic Indexes for Areas (SEIFA), Australia. Canberra: Australian Bureau of Statistics

[bib2] Australian Cancer Network Working Party (1997) The Pathology Reporting of Breast Cancer: A Guide for Pathologists, Surgeons and Radiologists. Sydney: Australian Cancer Network

[bib3] Australian Cancer Network Working Party (2001) The Pathology Reporting of Breast Cancer: A Guide for Pathologists, Surgeons and Radiologists. Sydney: Australian Cancer Network Working Party

[bib4] Barchielli A, Paci E, Giorgi D (1999) Recent trends of *in situ* carcinoma of the breast and mammographic screening in the Florence area, Italy. Cancer Causes Control 10: 313–317 10482490 10.1023/a:1008992903478

[bib5] Blanks RG, Moss SM, Patnick J (2000) Results from the UK NHS breast screening programme 1994–1999. J Med Screen 7: 195–198 11202586 10.1136/jms.7.4.195

[bib6] Choi WS, Parker BA, Pierce JP, Greenberg ER (1996) Regional differences in the incidence and treatment of carcinoma *in situ* of the breast. Cancer Epidemiol Biomarkers Prev 5: 317–320 8722225

[bib7] Dobson AJ, Kuulasmaa K, Eberle E, Scherer J (1991) Confidence intervals for weighted sums of Poisson parameters. Stat Med 10: 457–462 2028128 10.1002/sim.4780100317

[bib8] Ernster VL, Ballard-Barbash R, Barlow WE, Zheng Y, Weaver DL, Cutter G, Yankaskas BC, Rosenberg R, Carney PA, Kerlikowske K, Taplin SH, Urban N, Geller BM (2002) Detection of ductal carcinoma *in situ* in women undergoing screening mammography. J Natl Cancer Inst 94: 1546–1554 12381707 10.1093/jnci/94.20.1546

[bib9] Ernster VL, Barclay J, Kerlikowske K, Grady D, Henderson C (1996) Incidence of and treatment for ductal carcinoma *in situ* of the breast. JAMA 275: 913–918 8598618

[bib10] Estoesta J, Supramaniam R, Brassil A, Taylor R (2000) BreastScreen NSW Ten Year Statistical Report: 1988–98. Sydney: BreastScreen NSW

[bib11] Evans AJ, Pinder SE, Ellis IO, Wilson ARM (2001) Screen detected ductal carcinoma *in situ* (DCIS) overdiagnosis or an obligate precursor of invasive disease? J Med Screen 8: 149–151 11678555 10.1136/jms.8.3.149

[bib12] Giles G, Russell I, Reed R, Marr G, Kavanagh A (2001) *In situ* and small invasive breast cancer register in Victoria, 1988 to 1992: tumour characteristics and patient management. Aust N Z J Surg 71: 266–270 10.1046/j.1440-1622.2001.02100.x 11374473

[bib13] Kricker A, Armstrong B, Smith C, Bilous M, Camaris C, Mayer A, Psarianos T (1999) An audit of breast cancer pathology reporting in Australia in 1995. Br J Cancer 80: 563–568 10408867 10.1038/sj.bjc.6690392 PMC2362319

[bib14] Levi F, Te VC, Randimbison L, La Vecchia C (1997) Trends of *in situ* carcinoma of the breast in Vaud, Switzerland. Eur J Cancer 33: 903–906 9291813 10.1016/s0959-8049(97)00048-8

[bib15] Productivity Commission (2002) Report on Government Services 2002 http://www.pc.gov.au/gsp/2002/

[bib16] Shugg D, White VM, Kitchen PR, Pruden M, Collins JP, Hill DJ (2002) Surgical management of ductal carcinoma *in situ* in Australia in 1995. ANZ J Surg 72: 708–715 12534380 10.1046/j.1445-2197.2002.02532.x

[bib17] Silverstein MJ. (2003) The University of Southern California/Van Nuys prognostic index for suctal carcinoma *in situ* of the breast. Am J Surg 186: 337–343 14553846 10.1016/s0002-9610(03)00265-4

[bib18] Solin LJ, Recht A, Fourquet A, Kurtz J, Kuske R, McNeese M, McCormick B, Cross MA, Schultz DJ, Bornstein BA, Spitalier J-M, Vilcoq JR, Fowble BL, Harris JR, Goodman RL (1991) Ten-year results of breast-conserving surgery and definitive irradiation for intraductal carcinoma (ductal carcinoma *in situ*) of the breast. Cancer 68: 2337–2344 1657351 10.1002/1097-0142(19911201)68:11<2337::aid-cncr2820681102>3.0.co;2-r

[bib19] Thomson JZ, Evans AJ, Pinder SE, Burrell HC, Wilson ARM, Ellis IO (2001) Growth pattern of ductal carcinoma *in situ* (DCIS): a retrospective analysis based on mammographic findings. Br J Cancer 85: 225–227 11461081 10.1054/bjoc.2001.1877 PMC2364049

[bib20] Wärnberg F, Bergh J, Holmberg L (1999) Prognosis in women with a carcinoma *in situ* of the breast: a population-based study in Sweden. Cancer Epidemiol Biomarkers Prev 8: 769–774 10498395

[bib21] Zheng T, Holford TR, Chen Y, Jones BA, Flannery J, Boyle P (1997) Time trend of female breast carcinoma *in situ* by race and histology in Connecticut, USA. Eur J Cancer 33: 96–100 9071907 10.1016/s0959-8049(96)00371-1

